# Glycine Effect on the Expression Profile of Orphan Receptors GPR21, GPR26, GPR39, GPR82 and GPR6 in a Model of Inflammation in 3T3-L1 Cells

**DOI:** 10.3390/life12111687

**Published:** 2022-10-24

**Authors:** Rocío Alejandra Gutiérrez-Rojas, Karla Aidee Aguayo-Cerón, Cruz Vargas-De-León, Sandra Edith Cabrera-Becerra, Julio Cesar Almanza-Pérez, Fengyang Huang, Santiago Villafaña, Rodrigo Romero-Nava

**Affiliations:** 1 Escuela Nacional de Ciencias Biologicas del Instituto Politécnico Nacional, Ciudad de México 11340, Mexico; 2 Laboratorio de Señalización Intracelular, Sección de Estudios de Posgrado, Escuela Superior de Medicina del Instituto Politécnico Nacional, Ciudad de México 11340, Mexico; 3 División de Investigación, Hospital Juárez de México, Ciudad de México 07760, Mexico; 4 Laboratorio de Farmacología, Departamento de Ciencias de la Salud, DCBS, Universidad Autónoma Metropolitana-Iztapalapa (UAM-I), Ciudad de México 09340, Mexico; 5 Laboratorio de Investigación en Farmacología, Hospital Infantil de México Federico Gómez (HIMFG), Ciudad de México 06720, Mexico

**Keywords:** orphan receptors, glycine, inflammation, adipokines

## Abstract

Background: Chronic or low-grade inflammation is a process where various immune cells are recruited from the periphery into adipose tissue. This event gives rise to localised inflammation, in addition to having a close interaction with cardiometabolic pathologies where the mediation of orphan receptors is observed. The aim of this study was to analyse the participation of the orphan receptors GPR21, GPR39, GPR82 and GPR6 in a chronic inflammatory process in 3T3-L1 cells. The 3T3-L1 cells were stimulated with TNF-α (5 ng/mL) for 60 min as an inflammatory model. Gene expression was measured by RT-qPCR. Results: We showed that the inflammatory stimulus of TNF-α in adipocytes decreased the expression of the orphan receptors GPR21, GPR26, GPR39, GPR82 and GPR6, which are related to low-grade inflammation. Conclusions: Our results suggest that GPR21 and GPR82 are modulated by glycine, it shows a possible protective role in the presence of an inflammatory environment in adipocytes, and they could be a therapeutic target to decrease the inflammation in some diseases related to low-grade inflammation such as diabetes, obesity and metabolic syndrome.

## 1. Introduction

Inflammation is a local response to cellular injury, producing a host of chemical mediators that involves cytokines and other anti-inflammatory mediators [1,2]. A chronic, low-grade inflammatory state is a pathological feature involved in metabolic syndrome (MetS), non-alcoholic fatty liver disease (NAFLD), type 2 diabetes mellitus (T2DM) and cardiovascular disease (CVD) [3]. Inflammation is an important determinant of cardiometabolic dysfunction and increases the risk of T2DM, NAFLD and CVD associated with obesity [4]. Adipose tissue hypertrophy is associated with immune cell infiltration (macrophages and T cells) and a local pro-inflammatory state with the participation of cytokines such as TNF-α, IL-6 and IL-1β, inducing insulin resistance [5], thereby deregulating glucose and lipid metabolism in adipose tissue, skeletal muscle and the liver [2]. This hypertrophy process contributes to oxidative stress and the appearance of low-grade inflammation, increasing the activation of NF-κB (nuclear factor kappa light chain enhancer of activated B cells), TNF-α and leptin, while also decreasing IL-10 and AdipoQ [6]. Nutraceutical agents have been identified that participate in the reduction of low-grade inflammation [7,8].

Glycine is a non-essential amino acid found at high levels in plasma [9,10], but a decrease in these levels has been associated with low-grade inflammation in diseases such as obesity, type 2 diabetes mellitus and cardiovascular diseases [11,12,13]. Glycine decreases gene and protein expression of TNF-α and IL-6 in 3T3-L1 adipocytes [14,15] and inhibits activation of the NF-κB pathway by antagonistic effect of TNF-α1a receptor [16]. This amino acid modulates the expression of different receptors and could modulate the expression of some orphan receptors that have a role in pathologies related to the inflammatory process. According to the International Union of Basic and Clinical Pharmacology (IUPHAR), glycine as a ligand has different natural/endogenous targets, e.g., the glycine receptor (consisting of glycine receptor α1, α2, α3, α4 and β subunits), ionotropic glutamate receptors as a coagonist (GluN1, GluN2A, GluN2B, GluN2C and GluN2D), GPRC6 receptors and transporters, which move this compound across the lipid membrane (GlyT1 and GlyT2).

G-protein-coupled receptors (GPCRs) play a role in the regulation of physiological processes and represent approximately 30% of therapeutic targets that can be exploited [17,18]. The endogenous ligands of many GPCRs are yet to be identified, so they are collectively referred to as orphan GPCRs (oGPCRs) [19]. This kind of membrane receptor is of great interest because they could be possible therapeutic targets for the treatment of pathologies that involve low-grade inflammation [20]. However, the absence of known ligands significantly limits the experimental strategies available for the study of orphan receptor activation and signalling pathways [21,22]. Constitutive activity is observed when a GPCR produces spontaneous G-protein activation in the absence of an agonist [23], when the GPCR is overexpressed or when expression has decreased [24,25]. This is occasionally implicated in disease processes [26].

GPR21 is a rhodopsin-like orphan receptor, broadly expressed in different tissues and organs. This receptor shows constitutive activity through Gαq-type G-proteins, specifically Gαq and Gα15/16 [27]. Interestingly, GPR21 has been shown to be involved in the pathogenesis of insulin resistance, thus representing a potential new target for the treatment of type 2 diabetes and metabolic syndrome [28,29]. It has been suggested that GPR21 may coordinate macrophage pro-inflammatory activity and other cells such as adipocytes when there is obesity-induced insulin resistance [30].

GPR26 is an orphan GPCR without known endogenous ligands [31]. There is 95% sequence identity between human and mouse orthologs, indicating strong phylogenetic conservation of the protein structure and associated functional properties [32]. GPR26 is coupled to Gαs, which leads to an increase in cyclic AMP (cAMP) levels in target cells [33]. It is expressed in brain regions related to appetite control [34] and mood regulation [19]. It has been shown in animal models of metabolic syndrome that *GPR26* gene-expression levels are decreased in cardiac tissues [35], it suggests that GPR26 could play a role in low-grade inflammation.

GPR39 is another GPCR found in all vertebrates and is structurally homologous to the ghrelin receptor in the human foetal brain [36]. Until now, no endogenous peptide ligand has been discovered for GPR39 [37]. However, physiological concentration of Zn^2+^ has shown to activate GPR39 [38,39]. GPR39 activation induces signalling pathways through Gαq, Gαs, Gα11/12 and β-arrestin that regulates various cellular functions, such as survival, proliferation, differentiation and ion transport [38,40]. Ionic zinc is the only endogenous ligand for GPR39 identified [41,42]. However, synthetic ligands have been shown to be able to increase GPR39 signalling. It is thus possible that zinc is simply an enhancer and coactivator of another endogenous ligand yet to be identified [43]. Several studies have indicated that GPR39 interacts with the NF-κB signalling pathway [44,45] and could be involved in the low-grade inflammation process. In vitro evidence has demonstrated that GPR39 exhibits anti-inflammatory activity by reducing the expression of pro-inflammatory cytokines (IL-1β, IL-6) and enhancing anti-inflammatory cytokine production (IL-10) [44,46]. Orphan receptor *GPR39* gene expression is modulated in metabolic syndrome, which suggests that it may be involved in the development of this syndrome [35].

GPR82 has been classified as a class A orphan receptor that belongs to the group of chemoreceptors for adenosine-diphosphate-type receptors (P2Y12) [47]. This receptor is associated with a reduction in body weight, fat content and triglyceride levels [48]. Gene silencing of GPR82 mediated by siRNA in vivo decreased the values of systolic blood pressure and triglycerides, and increased HDL cholesterol, during the development of metabolic syndrome induced by fructose intake [49]. GPR82 is ubiquitously expressed, with the highest mRNA levels in the epididymis, testis and hypothalamus. This receptor shows conserved transcript and promoter structures, indicating that the GPR82 gene is functional in mice and humans [48].

GPR6 is a Gαs-coupled receptor that its highly expressed in the human striatum and hypothalamus [50]. It exhibits high constitutive activation of adenylyl cyclase, thereby increasing cAMP [51]. It is a rhodopsin-like receptor [52] and has been associated with the cannabinoid family because of its recognition of a sub-set of cannabinoid ligands [53]. It has been suggested that cannabidiol (CBD) acts as an inverse agonist at GPR6, indicating a potential therapeutic effect in Alzheimer’s disease and Parkinson’s disease [54]. There are patented imidazolidinethione and imidazodithiazole derivatives used as GPR6 inverse agonists, with potential use in the treatment of obesity [54,55].

3T3-L1 preadipocytes are typical cells frequently used in lipid metabolism research. In this study, we explored the gene expression of *GPR21*, *GPR26*, *GPR39*, *GPR82* and *GPR6* in this cell-type. Moreover, the effects of TNF-α and glycine on the modulation of these orphan receptors were investigated, suggesting participation in the low-grade inflammatory process.

## 2. Materials and Methods

### 2.1. Cell Culture

Fibroblasts differentiated into adipocytes from the 3T3-L1 cell line (ATCC, Manassas, VA, USA). For differentiation, the cells were cultured in 6-well plates (8 × 10^4^ cells per well) in DMEM/F12 (Gibco, Grand Island, NY, USA), supplemented with 10% fetal bovine serum (FBS, Gibco, Grand Island, NY, USA) and an antibiotic antimycotic solution (10,000 units penicillin, 10 mg streptomycin and 25 μg amphotericin B) (Sigma-Aldrich, St Louis, MO, USA). Adipocytes were maintained in a humidified atmosphere and CO_2_ (5%) at 37 °C. Fibroblasts were differentiated into adipocytes using DMEM supplemented with 10 mg/L murine insulin (Sigma-Aldrich, St Louis, MO, USA), 0.25 μM dexamethasone, (Sigma-Aldrich, St Louis, MO, USA) and 0.5 mM of methyl-isobutyl-xanthine (MIX, Sigma-Aldrich, St Louis, MO, USA). The medium was replaced every 48 h.

### 2.2. Experimental Design

Adipocyte treatments included: (1) control group (n = 3): cells without stimulus; (2) TNF-α group (n = 3): cells cultured with 5 ng/mL TNF-α for 60 min; (3) Gly group (n = 3): cells cultured with 10 mM Gly (60 min); (4) TNF + Gly group (n = 3): cells incubated with 5 ng/mL TNF-α for 30 min, followed by 10 mM glycine being added to the medium; and (5) Gly + TNF group (n = 3): cells pre-treated with 10 mM glycine for 30 min, followed by 5 ng/mL TNF-α being added to the medium. Finally, the adipocytes were lysed for RNA extraction 30 min after the last stimulus.

### 2.3. RNA Extraction and cDNA Synthesis

The RNA of adipocytes cultured in 6-well plates was extracted with 500 μL of guanidinium thiocyanate (TRIzol, Invitrogen), following the manufacturer’s instructions. The total RNA concentration and purity were quantified by a NanoPhotometer (Implen, Inc. Eastlake Village, CA, USA); the optical densities were evaluated at 260/280 nm and 260/230, and ratio of 1.8–2.2 indicated sufficient purity. RNA integrity was assessed on agarose gels (18 s and 28 s). Reverse transcription (RT) was performed using M-MLV Reverse Transcriptase (Invitrogen, Carlsbad, CA, USA), with 1000 ng of total RNA according to the manufacturer’s instructions. The cDNA was stored at −30 °C and then used to determine gene expression.

### 2.4. Quantitative Real-Time RT-qPCR

- *GPR21, GPR26, GPR39, GPR82, GPR6, TNF-α, adipoQ* and *36B4* (*Rplp0*) as the housekeeping gene were analysed by the Nano LightCycler System (Roche Diagnostics) (Table 1). RT-qPCR was performed using the FastStart Essential DNA Probes Master Mix (Roche Applied Science, Mannheim, Germany). Probes from Universal Probe Library were used (Table 1) (Roche Applied Science, Mannheim, Germany), with 0.3 µL of each primer (Oligo T4, Irapuato, Mexico) and 1000 ng of RNA. The reaction was performed in three steps. Step 1: 10 min preheating at 95 °C; step 2: 45 cycles of 15 s at 90 °C, 30 s at 60 °C and 15 s at 72 °C; step 3: cooling for 300 s at 40 °C. Relative changes in gene expression were determined using the 2^−ΔΔCt^ method [56].

### 2.5. Principal Component Analysis (PCA) of Existing Orphan Receptors Involved in Inflammatory and Anti-Inflammatory Processes

Principal component analysis (PCA) was performed using the gene expression variables. PCA was conducted using R statistical software version 4.1 with the R packages FactoMineR [57], factoextra [58] and ggplot2 [59]. We used the Kaiser–Meyer–Olkin (KMO) measure of sampling adequacy for the PCA; KMO values < 0.6 indicate inadequate sampling.

### 2.6. Statistical Analysis

The data were analysed by GraphPad Prism version 7 (Dotmatics, San Diego, CA, USA). The gene expression changes were assessed by one-way ANOVA, with the Tukey post hoc test (*p* < 0.05).

## 3. Results

### 3.1. Impact of Inflammatory and Anti-Inflammatory Environments on GPR21, GPR26, GPR39, GPR82 and GPR6 Gene Expression

**Figure 1:**
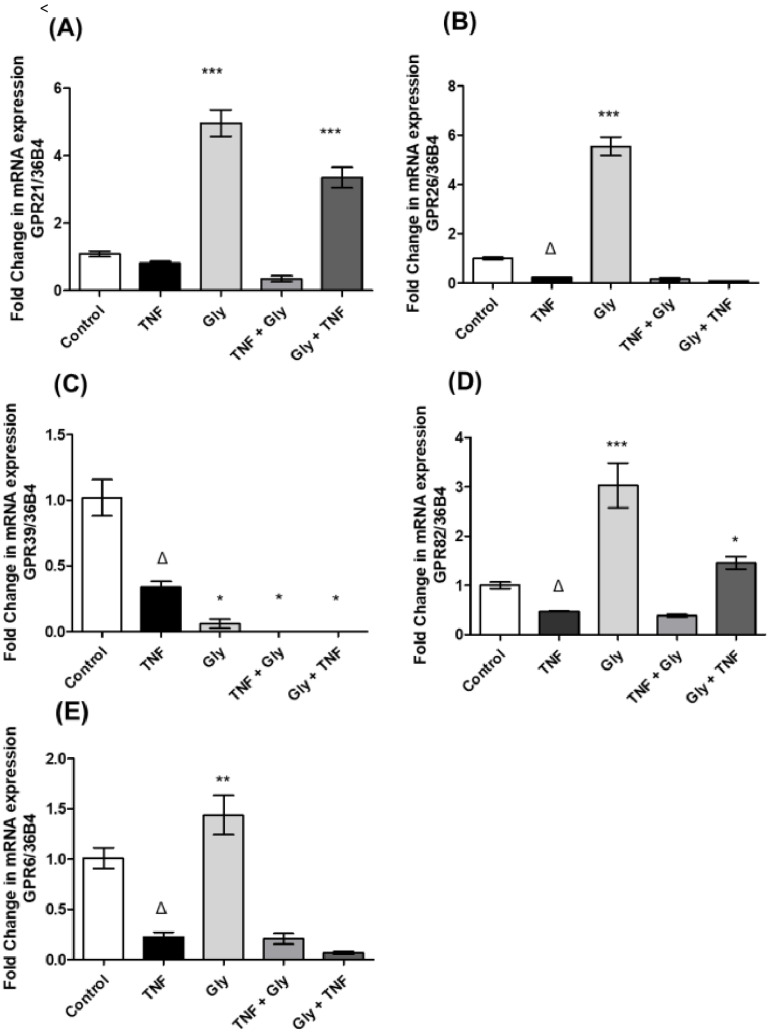
Orphan receptor gene expression in a pro-inflammatory environment. Control = control group (without stimulus), TNF = TNF-α group (TNF-α stimulus), Gly = glycine group (glycine stimulus), TNF + Gly = TNF-α stimulus before glycine stimulus, Gly + TNF = glycine pre-treatment before TNF-α stimulus. (**A**) *GPR21* gene expression; (**B**) *GPR26* gene expression; (**C**) *GPR39* gene expression; (**D**) *GPR82* gene expression; and (**E**) *GPR6* gene expression. The data are expressed as the mean ± standard error (n = 3). Normalised to the *36B4* housekeeping gene. * Significant difference between the TNF group and the other groups. * *p* < 0.05, ** *p* < 0.01, *** *p* < 0.001. ^Δ^ Significant difference between stimuli and control group. ^Δ^ *p* < 0.05.

TNF-α was used to create an inflammatory environment in 3T3-L1 adipocytes. Our results showed that *TNF-α* (one hour before RNA extraction) decreased the expression of *GPR26* (Figure 1B), *GPR39* (Figure 1C), *GPR82* (Figure 1D) and *GPR6* (Figure 1E), while *GPR21* (Figure 1A) expression remained unchanged compared to the unstimulated group. On the other hand, glycine (Gly) was used to create an anti-inflammatory environment in 3T3-L1 adipocytes. Cells were treated with glycine for about one hour, which increased the transcription levels of *GPR21* (Figure 1A), *GPR26* (Figure 1B), *GPR82* (Figure 1D) and *GPR6* (Figure 1E) respect to the control group. However, *GPR39* (Figure 1C) levels decreased further compared to the expression levels in the TNF-α-stimulated group.

### 3.2. Pre- and Post-Treatment with Glycine on Orphan Receptor Expression

Compared with the TNF-α group, treatment with glycine (60 min after TNF-α stimulus) showed a decrease of the gene expression of *GPR39* (Figure 1C), while the gene expression of *GPR21* (Figure 1A), *GPR26* (Figure 1B), *GPR82* (Figure 1D) and *GPR6* (Figure 1D) remained unchanged. Nevertheless, pre-treatment with glycine, followed by TNF-α stimulation, led to an increase in *GPR21* (Figure 1A) and *GPR82* (Figure 1D) expression, with a similar effect to treatment with glycine alone compared with the TNF-α group. For *GPR26* (Figure 1B), *GPR39* (Figure 1C) and *GPR6* (Figure 1E), no changes were noted.

### 3.3. Modulation of TNF-α and IL-6 Gene Expression by Glycine in Differentiated 3T3-L1 Adipocytes

**Figure 2:**
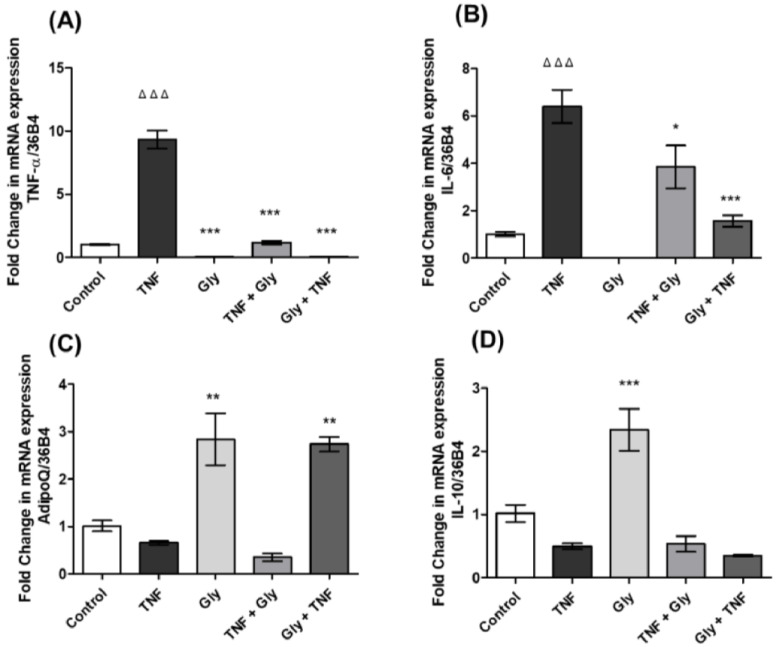
Cytokine gene expression. Control = control group (without stimuli), TNF = TNF-α group (TNF- α stimulus), Gly = glycine group (glycine stimulus), TNF + Gly = TNF-α stimulus before glycine stimulus, Gly + TNF = glycine pre-treatment before TNF-α stimulus. (**A**) *TNF-α* gene expression; (**B**) *IL-6* gene expression; (**C**) *AdipoQ* gene expression; and (**D**) *IL-10* gene expression. The data are expressed as the mean ± standard error (n = 3). Normalised to *36B4* housekeeping gene. * Significant difference compared between the TNF group and the other groups. * *p* < 0.05, ** *p* < 0.01, *** *p* < 0.001. ^Δ^ Significant difference compared between stimuli and control group. ^ΔΔΔ^ *p* < 0.001.

Our results showed that the inflammatory environment generated by TNF-α increased the expression of *TNF-α* (Figure 2A) and *IL-6* (Figure 2B) in 3T3-L1 cells respect to the control group, while stimulation with glycine decreased the expression of these two messengers compared to the group stimulated with TNF-α. On the other hand, we observed that treatment with glycine 30 min after stimulation with TNF-α also decreased the expression of *TNF-α* (Figure 2A) and *IL-6* (Figure 2B) when we compared to the group stimulated with TNF-α. In addition, the results showed that glycine pre-treatment before TNF-α stimulation also decreased the gene expression of *TNF-α* (Figure 2A) and *IL-6* (Figure 2B) respect to the inflammatory environment generated by TNF-α stimulation.

### 3.4. AdipoQ and IL-10 Gene Expression Changes by Glycine in 3T3-L1 Adipocytes

In this study, we evaluated the expression of anti-inflammatory cytokines such as adipoQ (Figure 2C) and IL-10 (Figure 2D) in 3T3-L1 cells. The results showed that stimulation with TNF-α decreased the expression of the messengers *AdipoQ* (Figure 2C) and *IL-10* (Figure 2D), while glycine increased this expression, when compared to the control group. On the other hand, we observed that treatment with glycine after creating an inflammatory environment by stimulation with TNF-α did not change the expression of these two anti-inflammatory cytokines, while pre-treatment with glycine before generating an inflammatory environment increased the expression of *AdipoQ* only (Figure 2C) respect to the group stimulated only with TNF-α; the expression of *IL-10* (Figure 2D) remained unchanged.

### 3.5. Principal Component Analysis

**Figure 3:**
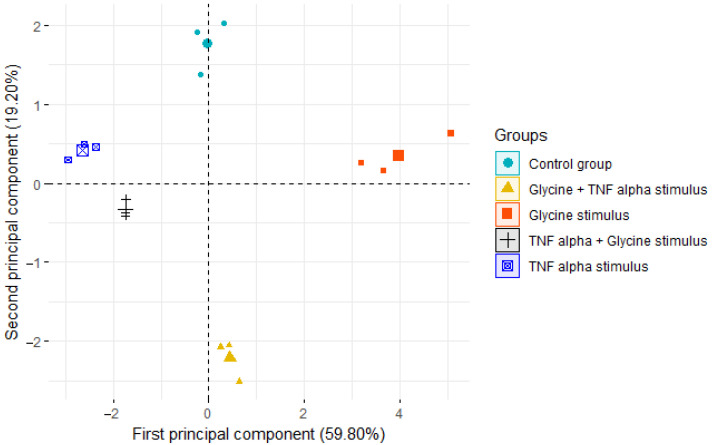
Biplot showing the distribution of groups according to the two first principal components. The largest symbol is the centroid of each cluster, and the other points are the subjects.

The Kaiser–Meyer–Olkin value of 0.63 was suitable for PCA. Subsequently, PCA was implemented to perform a dimensional reduction of nine gene expression variables. The first two principal components explained 79.0% of the variability. Table 2 shows the variable loading and correlation coefficients for the principal component scores. These components were integrated as follows: first component: *GPR21, GPR26, GPR82, GPR6, TNF-α, adipoQ, IL-6* and *IL-10*; second component: *GPR21, GPR39* and *GPR6*. In Figure 3, a biplot of gene expression data is shown. The PCA shows that five clusters formed, i.e., the five treatment groups of this study. Using PCA, we have shown that orphan receptor gene expression, and the pro- and anti-inflammatory effects of TNF-α and glycine, can be described using a mathematical technique (PCA) to further enhance the field’s knowledge and understanding of the role of orphan receptors in the inflammatory process.

In Figure 3, the PCA showed that the samples within each group were very similar to each other, and that the groups were different from each other. This integral analysis of gene expression allowed us to observe that the control group and the group with glycine + TNF-α stimulation were dissimilar.

Table 2 shows that GPR21, GPR26, GPR82, GPR6, AdipoQ and IL-10 of the first component have positive loading; it is well known that AdipoQ and IL-10 are anti-inflammatory, so GPR21, GPR26, GPR82 and GPR6 could have a similar role. GPR39, TNF-α and IL-6 have negative loading, TNF-α and IL-6 are known to be pro-inflammatory, so GPR39 could have a pro-inflammatory role. In the second component there is no structure in the loading that allows us to suggest a role in its function.

## 4. Discussion

Adipose tissue is regarded as a mere fat-store, with few active functions. However, this tissue has attracted considerable scientific interest, because it has recently been demonstrated that, in addition to regulating body fat and nutritional homeostasis, adipose tissue secretes a wide range of adipocytokines involving the participation of different membrane receptors and intracellular signalling pathways [60]. Our results showed that *GPR21, GPR26, GPR39, GPR82* and *GPR6* are expressed in 3T3-L1 cells during adipogenesis, with higher gene expression of the mRNA of *GPR26* and *GPR39,* while *GPR82* showed the lowest gene expression when compared to the expression of *GPR21*. *GPR6* showed gene expression like *GPR21*. Until now, only expression of the orphan receptor GPR39 has been determined in 3T3-L1 cells during the adipogenic process [61,62]. Understanding adipogenesis, i.e., the process of adipocyte development, may provide new alternatives to the treatment of obesity and metabolic diseases. Adipogenesis is controlled by coordinated actions of lineage-determining transcription factors and epigenomic regulators, in addition to the participation of receptors and their intracellular signalling pathways [63]. We evaluated the direct actions of TNF-α and glycine in cultured adipocytes. As shown in Figure 2, treatment of mouse 3T3-L1 cells with TNF-α reduced the expression of all orphan receptors in our study. We also demonstrated the ability of glycine to reverse these orphan receptor expression changes, showing an increase in *GPR21*, *GPR26*, *GPR82* and *GPR6*. In contrast, treatment with glycine led to decreased gene expression of *GPR39*, suggesting that the increase in the expression levels of *GPR21* and *GPR82* could confer a protective effect, while decreased expression levels of *GPR39* presented a negative correlation with the glycine stimulus. Recent studies have shown that treatment with TNF-α reduces the expression of different G-protein-coupled receptors in human cells [64]. There is no evidence that TNF-α decreases the expression of these orphan receptors, nor that glycine increases the expression of *GPR21*, *GPR26*, *GPR82* and *GPR6* in mature adipocytes (3T3-L1 cells).

The present findings demonstrate the ability of glycine pre-treatment to induce early-response gene expression of *GPR21* and *GPR82* in response to an inflammatory stimulus, showing the possible participation of these two receptors in the modulation of the inflammatory response in 3T3-L1 cells during adipogenesis by generating an anti-inflammatory environment before an inflammatory stimulus. However, treatment with glycine after generating an inflammatory environment did not reverse the expression of any orphan receptor evaluated in this study in 3T3-L1 cells. GPCRs have attracted a great deal of interest owing to their numerous physiological and pathological roles in transducing signals through the activation of heterotrimeric G proteins. Most methods used to identify GPCRs assess specific expression profiles and distinct signal transduction pathways, which change due to the addition of external stimuli that are considered potential ligands.

Glycine represses the expression of pro-inflammatory cytokines such as TNF-α and IL-6 in Kupffer cells and stimulates the anti-inflammatory response by increasing the secretion of IL-10 [65]. This amino acid has been shown to have anti-inflammatory properties both in vivo and in vitro [10,14,66,67]. Our study showed that TNF-α increased the gene expression of *TNF-α* and *IL-6*, while decreasing the expression of *AdipoQ* and *IL-10*, generating an environment characteristic of an inflammatory process.

Glycine decreased *TNF-α* and *IL-6* gene expression in 3T3-L1 cells respect to the TNF-α-stimulated group; it was evident that the use of glycine after and before an inflammatory stimulus decreased the expression of these genes that are characteristic of a pro-inflammatory process. Glycine also increased the expression of *AdipoQ* and *IL-10* respect to the control group, reversing the effect produced by stimulation with TNF-α. Some studies have shown that the consumption of glycine favours a protective effect against inflammatory events, either due to infections or mechanisms that generate an inflammatory environment. Glycine has an important role in the regulation of gene expression [68], protein configuration, protein activity and several biological functions [69]. Accumulating evidence suggests that glycine protects various cells from inflammatory environment and oxidative stress [70,71], and attenuates oxidative stress and inflammation in a mouse model [72]. Glycine is considered an amino acid with anti-inflammatory and immunomodulatory effects in organisms and various types of cells [73]. It acts as a secretagogue for GLP-1 [74], insulin and glucagon [75]. Glycine supplementation in humans (5 g/day or 0.1 g glycine/kg/day for 14 days) improves insulin response and glucose tolerance in obese patients [76]. In animal models, it has been shown that glycine consumption decreases synovial hyperplasia and oedema in joints and prevents the infiltration of inflammatory cells [77]. In addition, glycine decreases the mRNA expression of pro-inflammatory cytokines such as *TNF-α* and *IL-6* [14,66] and increases the mRNA and protein levels of anti-inflammatory cytokines such as *AdipoQ* and *IL-10* in 3T3-L1 cells [15,78,79].

The PCA showed that the positive loading of GPR21, GPR26, GPR82 and GPR6 in the first component suggests that they have an anti-inflammatory function, and the negative loading of GPR39 suggests that it has a pro-inflammatory role.

Our current work showed the effect of glycine on the expression of orphan receptors GPR21, GPR26, GPR39, GPR82 and GPR6 in response to anti- or pro-inflammatory stimuli, and the consequences of these changes in the inflammatory or anti-inflammatory processes. PCA is a tool to discover correlations in an unbiased manner, as it helps us to identify synergistic processes that occur in biological systems and readily pinpoint parallel, independent processes, such as orphan receptor expression in pro- and anti-inflammatory environments. These results corroborate the inconclusive data found for the gene expressions of orphan receptors and their association with an inflammatory environment.

## 5. Conclusions

In conclusion, our results suggests that GPR21 and GPR82 are modulated by glycine, showing a possible protective role in the presence of an inflammatory environment in ad-ipocytes. On the other hand, PCA analysis shows that GPR21, GPR26, GPR82 and GPR6 have an anti-inflammatory function, while GPR39 has a pro-inflammatory role. These orphan receptors are expressed in adipocytes and could be considered as pharmacological targets in diseases related to low-grade inflammation such as diabetes, obesity and metabolic syndrome.

## Figures and Tables

**Table 1:** Primer design for *Mus musculus*.

Gene Symbol	Forward Primer	Reverse Primer	Probe (Roche)	Accession Number
Housekeeping				
*36B4*	F: 5′-AAGCGCGTCCTGGCATTGTCT-3′	R: 5′-CCGCAGGGGCAGCAGTGGT-3′	72	NM_007475.4
Genes of interest				
*GPR21*	F: 5′-GAACTCCACCTGGGATGGTA-3′	R: 5′-GTAGCCCAGTGCCAGAAGAC-3′	46	NM_177383.4
*GPR26*	F: 5′-GCCAGAGCAAAGGGAGGT-3′	R: 5′-AGGCAATGGTGGCAGTTATT-3′	10	NM_173410.3
*GPR39*	F: 5′-CGGCGCAGTAACTCTTCC-3′	R: 5′ -GGCCTCAGTCTGAAAAGTGC-3′	74	NM_027677.2
*GPR82*	F: 5′-GGAACAGAAAATATGACCTGATTCAT-3′	R: 5′- GAGGGCCTAGCACATAGCAA ′	36	NM_175669.4
*GPR6*	F: 5′-ACATGCCAGCCTTTGGTG-3′	R: 5′-GCACCACTGACACCTCAAGA-3′	40	NM_199058.2
*TNF-* *α*	F: 5′-TCTTCTCATTCCTGCTTGTGG-3′	R: 5′-GGTCTGGGCCATAGAACTGA -3′	49	NM_001278601.1
*IL-6*	F: 5′-ACAAAGCCAGAGTCCTTCAGA-3′	R: 5′-TGGTCCTTAGCCACTCCTTC-3′	78	NM_001314054.1
*AdipoQ*	F: 5′-GGCTCTGTGCTCCTCCATCT-3′	R: 5′-AGAGTCGTTGACGTTATCTGCA-3′	1	NM_009605.5
*IL-10*	F: 5′-CCCTGGGTGAGAAGCTGAAG-3′	R: 5′-GGGGAAGAACGCATCTGCTA-3′	30	NM_010548.2

**Table 2:** Results from the PCA on gene expression. For the first principal component, all variables had a correlation greater than 0.60, except GPR39. For the second component, GPR21, GPR39 and GPR6 had a moderate correlation.

Gene Expression	First Component Loading (Correlation)	Second Component Loading (Correlation)
*GPR21*	0.328 (0.760 **)	0.431 (0.566 *)
*GPR26*	0.367 (0.850 **)	−0.190 (−0.250)
*GPR39*	−0.058(−0.136)	−0.589 (−0.773 **)
*GPR82*	0.417 (0.966 **)	0.018 (0.024)
*GPR6*	0.334 (0.775 **)	−0.466 (−0.612 *)
*TNFα*	−0.280 (−0.648 **)	−0.169 (−0.222)
*AdipoQ*	0.359 (0.832 **)	0.305 (0.400)
*IL-6*	−0.357 (−0.829 **)	0.010 (0.013)
*IL-10*	0.367 (0.851 **)	−0.302 (−0.394)

* *p* < 0.05, ** *p* < 0.001.

## Data Availability

The study did not report any data.
